# Technical and Environmental Viability of a Road Bicycle Pedal Part Made of a Fully Bio-Based Composite Material

**DOI:** 10.3390/ma14061399

**Published:** 2021-03-13

**Authors:** David Hernández-Díaz, Ricardo Villar-Ribera, Ferran Serra-Parareda, Rafael Weyler-Pérez, Montserrat Sánchez-Romero, José Ignacio Rojas-Sola, Fernando Julián

**Affiliations:** 1 Serra Húnter Programme, Department of Engineering Graphics and Design, Polytechnic University of Catalonia, 08222 Terrassa, Spain; 2 Department of Engineering Graphics and Design, Campus Manresa, Polytechnic University of Catalonia, 08242 Manresa, Spain; 3 LEPAMAP Research Group, University of Girona, 17003 Girona, Spain; ferran.serrap@udg.edu; 4 Department of Strenght Materials and Structural Engineering, Polytechnic University of Catalonia, 08222 Terrassa, Spain; rafael.weyler@upc.edu (R.W.-P.); montserrat.sanchez@upc.edu (M.S.-R.); 5 Department of Engineering Graphics, Design and Projects, University of Jaén, 23071 Jaén, Spain; jirojas@ujaen.es; 6 Design, Development and Product Innovation, Department of Organization, Business, University of Girona, 17003 Girona, Spain; fernando.julian@udg.edu

**Keywords:** natural-fibre composites, green composites, biopolymers, ecological product design, mechanical properties, life cycle assessment

## Abstract

Glass fibre is the most widely used material for reinforcing thermoplastic matrices presently and its use continues to grow. A significant disadvantage of glass fibre, however, is its impact on the environment, in particular, due to the fact that glass fibre-reinforced composite materials are difficult to recycle. Polyamide 6 is an engineering plastic frequently used as a matrix for high-mechanical performance composites. Producing polyamide monomer requires the use of a large amount of energy and can also pose harmful environmental impacts. Consequently, glass fibre-reinforced Polyamide 6 composites cannot be considered environmentally friendly. In this work, we assessed the performance of a road cycling pedal body consisting of a composite of natural Polyamide 11 reinforced with lignocellulosic fibres from stone-ground wood, as an alternative to the conventional glass fibre-reinforced Polyamide 6 composite (the most common material used for recreational purposes). We developed a 3D model of a pedal with a geometry based on a combination of two existing commercial choices and used it to perform three finite-element tests in order to assess its strength under highly demanding static and cyclic conditions. A supplementary life cycle analysis of the pedal was also performed to determine the ecological impact. Based on the results of the simulation tests, the pedal is considered to be mechanically viable and has a significantly lower environmental impact than fully synthetic composites.

## 1. Introduction

Presently, 95% of all reinforced composites contain glass fibre, generating more than 7 billion dollars annually [1,2]. Composed of minerals and artificially synthesized, glass fibre enhances the mechanical properties of polymer matrices and makes composites lighter and stronger than their metal-based counterparts. These advantages have led to its widespread use in the automobile, aeronautic, and sports production industries, among others [3]. Ongoing research is aimed at improving the mechanical properties of glass fibre-based composites with supplementary reinforcing materials. These new materials, referred to as “hybrid composites” because they contain two or more reinforcements, are expected to expand the application field of composites and increase the use and consumption of carbon and glass fibres. However, glass fibre-based composites raise several concerns regarding safety risk assessment and environmental sustainability. Firstly, glass fibre is considered a skin irritant material that can lead to contact dermatitis when handled [4,5]. Furthermore, several acute respiratory health effects when glass fibre is inhaled have been reported, including asthma, chronic airflow obstruction, and increased risk of respiratory tract cancers [6,7,8,9]. These considerations must be considered in the manufacturing and recycling processes. Secondly, glass fibre-based composites are not environmentally friendly and are not easily recyclable [10,11,12]. The challenges associated with recycling glass fibre-based composites have been addressed following two approaches: by developing new, more effective recycling procedures, and by replacing composites based on mineral fibres, such as glass, with natural fibre-reinforced polymers (NFRPs). The present research addresses this last approach.

Natural fibres are less expensive, lighter, and less dense than synthetic fibres such as glass or carbon; hence, they are more attractive economically [13]. These advantages led to the commercialisation of synthetic fibre-reinforced polymers (SFRPs), previously based largely on metallic materials. Natural fibres used to reinforce thermoplastic matrices are less abrasive during material processing, provide better surface finishing with injection moulding, are biodegradable and have minimal health impacts, and are derived from abundant renewable sources [14,15]. However, large-scale use of NFRPs is still considered a challenge in terms of manufacturing, especially due to the anisotropic and heterogeneous nature of this type of material [16]. Replacing synthetic fibres with natural fibres is currently regarded as an evolutionary step, an opportunity to improve mechanical performance in reinforced composites, and a promising pathway to greater environmental sustainability. For these reasons, there is increasing interest among scientists to expand on their potential uses and benefits.

Thermoplastic matrices have been reinforced with fibres from cellulose-rich plants such as flax, hemp, jute, kenaf, and sisal [17]. Such fibres have several environmental advantages over synthetic fibres, but they also have some disadvantages, particularly in terms of water uptake and mechanical impact strength. A significant amount of research in this field has focused on assessing these two properties in NFRPs to gain a better understanding of the influence of certain variables. The hydrophilicity of some plant fibres and their non-cellulose components has been cited as the greatest hindrance to using NFRPs in wet outdoor environments or under mechanically demanding conditions [14]. Chemically, a high cellulose content may be counterproductive and hinder the dispersion of fibres in thermoplastic matrices [18]. The decreased impact strength of NFRPs relative to synthetic fibre-reinforced composites has been attributed to increased fibre agglomeration, increased polymer stiffness upon reinforcement, increased number of stress concentrators around fibre ends, and contrasting hydrophilicity between the natural fibres and the matrix [19]. It has been shown that the mechanical strength of NFRPs can be substantially improved by adding an appropriate amount of a compatibilizer [19] for better dispersion of reinforcing fibres in the matrix [20], increased adhesion between the two [21,22] and more efficient matrix-to-fibre transfer of mechanical stress [23].

Replacing synthetic fibres with natural fibres is one approach, but it is not the only way to obtain more sustainable composites. Another approach is to replace petroleum-based plastic matrices with others from renewable sources such as proteins, polysaccharides, lipids and polyesters from plants or microbes—or with materials obtained from renewable sources [24]. These plastics are known as “bio-based polymers” and have gained much scientific interest due to the increasing demand for, and scarcity of, oils [25], and also to the harmful environmental impacts of conventional plastics [26,27]. Bio-based polymers, however, are still not yet used widely [28] due to their poor mechanical performance relative to synthetic plastics [29]. Despite this, they are becoming increasingly common in biomedical, packaging, agricultural, and food applications [30]. As with conventional plastics, one approach to improve the mechanical properties of bio-based matrices is to insert reinforcing fibres or fillers. Plastics containing natural as opposed to synthetic reinforcements are an attractive option for environmentally friendly engineering. Some composites of this type have been shown to possess good mechanical, thermal, and electrical properties [31]. Additionally, bio-based production costs have gradually reduced to levels similar to those of conventional synthetic plastics [32,33].

In this work, we adopted a case study methodology to assess mechanical performance and industrial viability in a bicycle pedal consisting of a fully bio-based composite compared to a pedal made from conventional materials. The composite materials examined were bio-based Polyamide 11 reinforced with lignocellulosic fibres from stone-ground wood (SGW) and a synthetic polyamide reinforced with glass fibre. A 3D model of the pedal was generated by using a hybrid design based on two different commercial brands. The main piece of the modelled pedal was assigned the bio-based composite, and its mechanical performance was assessed under static and cyclic loads through computer simulations. In order to assess the sustainability of the production of the bio-based material in relation to its commercial counterpart, which requires careful investigation [34,35], both were subjected to a supplementary life cycle analysis (LCA) to assess their environmental impact in terms of raw materials, transport, production, use, and end of life [36]. The carbon footprint and energy consumption for the two materials were also assessed.

The primary aims of this work were to validate theoretically the technical viability of a fully bio-based product and to quantify the environmental benefits of its use considering a holistic life cycle. The novelty of this work is the assessment of mechanical performance of an environmentally friendly composite material, and an approach to advantageously replace composites based on technical, non-generalist matrices. Due to the fact that one of the greatest weaknesses of natural fibre-reinforced composites is their mechanical properties, we examined their mechanical performance under static conditions; we also performed a novel investigation of the fatigue behaviour of the bio-based product under cyclic loadings. In studying natural reinforced composites, it is common to analyse the thermomechanical properties of the materials to predict their behaviour, but not to perform fatigue tests; therefore, analysing the fatigue behaviour of the bio-based composite is also an innovative contribution of the present research. We consider this study extension essential to assure designers and engineers that these more sustainable and environmentally friendly materials can be reliably used in new products and applications. Consequently, the hypothesis of this research is that, for some applications, bio-based composites can replace synthetic composites, without a loss in performance and offering several important environmental benefits.

The remaining sections of this paper are structured as follows: Section 2 describes the methodology and specifies the materials used, Section 3 presents the test results, and Section 4 reports conclusions from the test results.

## 2. Materials and Methods

The experimental design began with selecting the specific test target (viz., the test case). For this purpose, we examined products used by sports professionals typically manufactured from composites reinforced with synthetic fibres, where the plastic matrix is an engineering plastic that can withstand relatively high mechanical stress. The aim was not to simply select a conventional product but to confirm that a bio-based composite was a viable choice for developing industrial products capable of fulfilling relatively demanding application requirements. The product selected for the analysis was an automatic pedal for road bicycles.

In terms of design, the geometry and constituent material of a product can influence factors such as its processability and its disposal at the end of its life cycle [37,38]. Therefore, any decisions made in these respects should be based on careful economic, social, and environmental analyses [39,40]. The pedal studied here was a hybrid of two commercial models: Kéo Classic 3 from Look Cycle Group (Nevers, France), and Xpresso 4 from Time Sport International (Voreppe, France). Both were scanned on a Crysta Apex 544 3D coordinate measuring machine from Mitutoyo (Takatsu-ku, Japan). The pedal geometry was suitable for use with the Kéo Classic cleat from Look Cycle Group or a compatible model. A hybrid design was selected based on the primary aim of the study, which was not to assess brands for performance but rather to use a pedal geometry as close as possible to that of commercial models in order to examine the influence of test variables other than the manufacturing material. The pedal was modelled using the SolidWorks 2020 software from Dassault Systèmes (Vélizy-Villacoublay, France). Smooth integration of the test component into the system was checked by modelling the pedal itself as well as the entire assembly.

The bio-based material used in the simulations was Polyamide 11 (PA11) reinforced with 60 wt% natural fibres from SGW. In a previous study, this bio-based composite was found to have similar properties to polypropylene reinforced with 20–30% of glass fibre (S-Glass and C-Glass); however, the increased density of the bio-based polyamide resulted in slightly worse mechanical performance in the natural composite [41]. The synthetic alternative used for comparison was a composite consisting of a Polyamide 6 matrix reinforced with glass fibre (PA6 + GF), which is the most extensively used material in non-professional road bicycle pedals. The proportion of reinforcing fibres used in composites differs widely among manufacturers, accounts for 10–50% of the total pedal weight. Increasing the proportion results in a linear increase in composite tensile strength [42], but also in increased density—which is important since pedals should be as lightweight as possible. Due to the large number of commercial PA6 + GF references available, we used the average values for the technical parameters for each grade from a well-known materials library [43]. Table 1 lists the macroscopic mechanical properties of the materials considered. The variables $\sigma_{t}^{C}$ and $\sigma_{f}^{C}$ in the table denote the ultimate tensile strength and bending strength, respectively; $E_{t}^{C}$ and $E_{f}^{C}$ are the tensile and bending elastic modulus, respectively; and $\varepsilon_{t}^{C}$ and $\varepsilon_{f}^{C}$ are the elongation at break under tensile and bending loads, respectively. Although the tensile and flexural properties of the bio-based composite differed markedly from the average values for the synthetic composites, according to the consulted material library, they were within the ranges of values of some glass fibre-reinforced PA 6 composites, as can be seen in Table 2. The tensile and flexural strengths of the bio-based material were in fact similar to those for some low-grade composites consisting of PA6 reinforced with up to 30% of glass fibre.

Once the characteristics of the bio-based composite were assigned to the 3D prototype for the bio-based pedal, three finite-element numerical analyses were performed using SolidWorks (v.2020, Dassault Systèmes, Vélizy-Villacoublay, France). A first static test was performed to simulate cleat engagement. No maximum force for this event had seemingly been reported, where the cyclist, with both hands on the handlebar and one foot on the ground—or already on the other pedal—fits the cleat on the pedal. This led us to assume a hypothetical abuse of the pedal by applying a force of 500 N to engage the cleat. A second static test was performed to simulate the highest effective force acting on the pedal during pedalling. The force exerted by a cyclist on a pedal changes over each rotation cycle of the connecting rod and can be separated into an effective pedalling force and an ineffective force along the direction of the rod [44,45]. A number of researchers have measured the applied or effective force during pedalling with the goal of optimizing cycling technique and performance [44,45,46,47,48,49,50,51]. The highest value reported of 1294 N was obtained in a professional performance test under sprint conditions [44]. We thus used a load of 1300 N to simulate this limiting situation. We also considered examining the scenario in which cyclists mount or dismount a bicycle by placing their whole weight on a pedal. This is uncommon with non-stationary bicycles and, should it occur, part of the cyclist’s weight would fall on the handlebar. A bicycle user weighing 120 kg or less would be unable to apply a force greater than 1300 N, therefore we assumed this scenario to be addressed by the previous test. The last finite-element test was a fatigue analysis which involved loading the pedal with a force of 165 N for 10^6^ cycles. Considering a pedalling cadence of 80 rpm, the force value was obtained from the average power value measured for professional cyclists on a mountain stage of the Tour of Italy, which was 235W [52]. Whereas the fatigue S–N curve for a bio-based composite is not available in the literature, it has been reported for a Polyamide 6.6 composite reinforced with 40% of glass fibre (PA6.6 + 40%GF) [53,54]. Since the curve of interest was identical with that for non-reinforced PA6.6—the ductile matrix prevails in the fatigue behaviour of composites with strong fibre–matrix bonds [55]—and PA11 has much better fatigue properties than PA6.6 [56,57], the behaviour of the bio-based composite was conservatively assumed to be similar to that of the petroleum-derived composite, which limited the scope of the study.

The environmental and commercial implications of the bio-based material were compared with those of the commercial composite in an LCA by using the sustainability module in SolidWorks 2020 under the assumption that the pedal would be manufactured and recycled in Europe. The geometry used was that of the hybrid pedal’s main body, which was assumed to consist of the materials shown in Table 1.

## 3. Results

### 3.1. Definition of the Case Study

As discussed above, the test case was the main body of an automatic bicycle pedal. This type of pedal enables more efficient pedalling than the classical model by virtue of a pedal–shoe interface that results in more effective pedalling force during each rotation of the connecting rod. Automatic pedals are widely used among professional road cyclists and extensively used by recreational bikers. The cyclist’s shoe fits the pedal through a piece called a “cleat” that is screwed onto the shoe. Generally, the pedal components in contact with the cleat are made of a composite reinforced with synthetic fibres, whereas the cleat consists of non-reinforced thermoplastic material (e.g., polyacetal in the Kéo Look model cleat).

**Figure 1:**
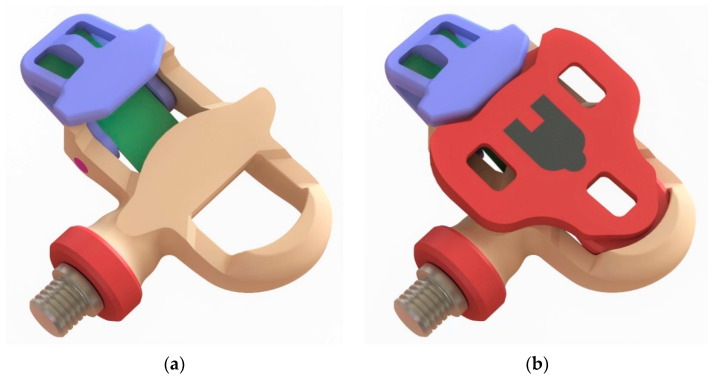
Proposed hybrid pedal design. (**a**) Cleat off; (**b**) cleat on.

**Figure 2:**
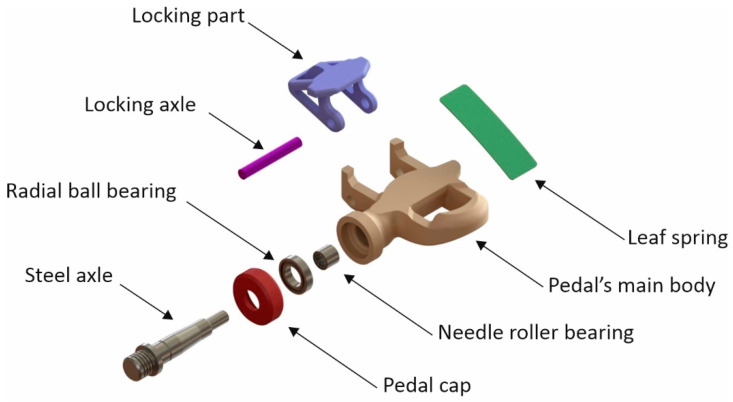
Exploded view of the pedal.

Figure 1 shows the hybrid pedal with the cleat off and on. Although mechanical computations were based on professional performance data, the target user was an amateur cyclist. The cleat was designed to be compatible with the Kéo Look model, which is something usual among manufacturers of other brands. An automatic pedal is comprised of the following components (Figure 2): main body; cleat-pedal locking part; steel axle, which is screwed to the connecting rod and receives pedalling forces via two bearings; and a carbon fibre sheet that acts as a spring to hold the main body-lock pair together. The cyclist’s shoe can be easily fitted on the pedal by placing the cleat front over the pedal hole and pressing slightly upwards to have the cleat retained. Conversely, rotating the foot to move the heel away from the bicycle’s vertical plane causes the spring to give and allows the foot to be detached from the pedal.

Unlike commercial models, the main body of the hybrid pedal consisted of a bio-based PA11 + 60% SGW composite rather than glass fibre-reinforced PA6 as in amateur pedals. The main body of an automatic pedal is the component under the greatest loading during pedalling; therefore, it was selected as the component to be simulated. The locking part could be made from the same material; in addition, all other components were made of the same materials as those used in automatic pedals for amateur cycling.

### 3.2. Analysis of the Test Case

As stated in Section 2, the finite-element tests were conducted using SolidWorks 2020 (specifically, with the simulation module available in the premium version). Two different static scenarios were considered: cleat engagement and pedalling. A third scenario was examined in order to assess the fatigue effects on the pedal’s main body which are produced by the cyclical loads applied during pedalling. Mechanical analyses were extended to the main body and steel axle (i.e., the element conveying loads), in order to simulate a more realistic contour condition as the axle is a deformable element over which the loads are distributed. In the static tests, the pedal was assumed to be a fixed element, and motion in the engagement zone was restricted. Loads were uniformly distributed over the surfaces of the pedal’s main body in contact with the cleat at the time a load was applied by the user. Both static analyses used a 10-node quadratic tetrahedral element mesh, with 75,328 elements and an average size of 1.667 ± 0.0833 mm. Precision was improved by refining the mesh in those zones under increased solicitation (viz., 16 sides of the pedal body geometry). The aspect ratio was greater than 3 in more than 98.3% of the elements. The outputs of the static finite-element tests were Von Mises stress (MPa), net displacement (mm), and strains (%), all with their corresponding colour maps on the 3D model.

**Figure 3:**
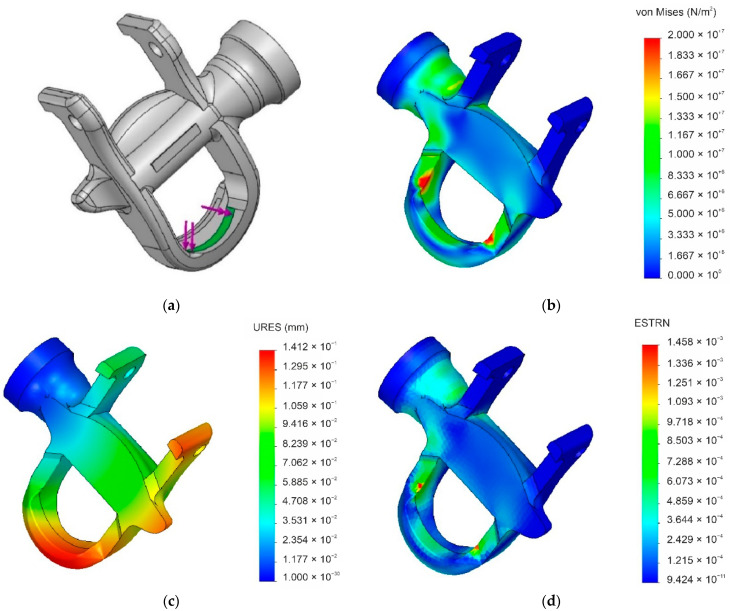
Graphic output summaries of the finite-element analysis of a PA11 + 60% SGW pedal body during cleat engagement under extreme use conditions. (**a**) Loaded surface; (**b**) Von Mises stress; (**c**) net displacement; (**d**) percent strain.

Engaging the cleat requires the cyclist to apply a slight force over a highly specific zone in the front of the pedal body (Figure 3a). When the cleat comes into contact with the pedal locking part, the spring gives and shifts the piece backwards. Once the cleat is properly placed on the pedal, the locking part returns to its initial position to ensure coupling of the cleat–pedal pair. The sheet acting as a spring during engagement of the cleat allows a relative movement between both components, reducing the stress analysis to the pressure exerted by contact between the cleat and the pedal’s body. Although the cleat can be engaged by applying a minimal force, it can be difficult for cyclists not used to automatic pedals to perform this action, and can even cause them to fall; in extreme situations, this can generate considerable loads on the surface of the pedal’s main body in contact with the cleat at the beginning of the engagement process. As no study has evaluated the effect of the load applied during the engagement operation, we used a conservative load of 500 N to represent a scenario of extreme misuse. Figure 3 shows the results of the finite element analysis in the engagement scenario between the cleat and the pedal. The maximum Von Mises stress obtained was 30.05 MPa, which is approximately half of the tensile strength of the composite, while the maximum displacement was 0.14 mm. The results indicate that there is no possibility of breakage of the piece under the conditions of analysis. Similarly, the small deformation values show that the bio-based composite has sufficient rigidity and the deformations that occur will not affect the functioning of the part.

**Figure 4:**
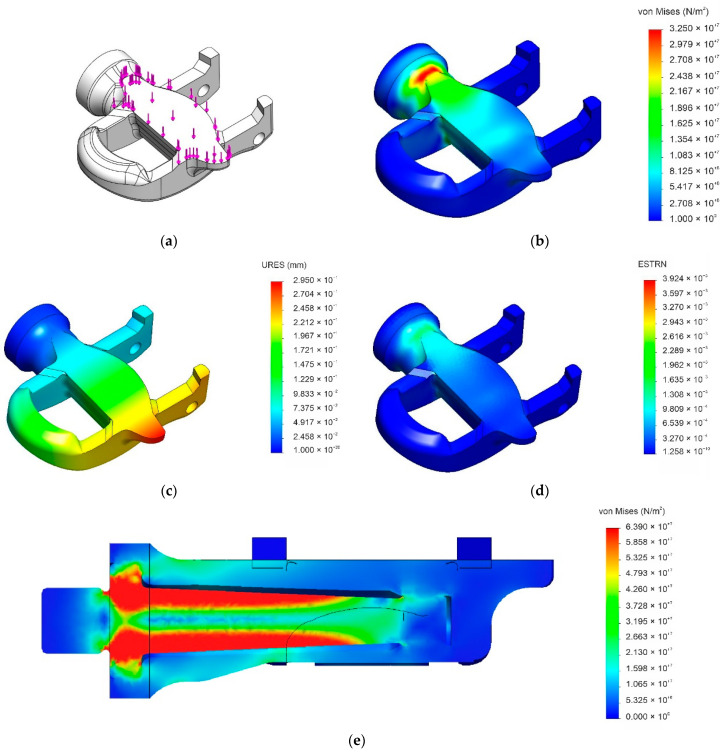
Graphic output summaries of the finite-element analysis of a PA11 + 60% SGW pedal body during pedalling under a force of 1300 N. (**a**) Loaded surface; (**b**) Von Mises stress; (**c**) net displacement; (**d**) percent strain; (**e**) effort transmission to axle.

The loaded surface differs between the cleat engagement and pedalling operations because, in the latter, the cleat is in contact with the central part of the pedal’s main body. Due to the fact that the cleat is anchored to the pedal, the surface over which the force is exerted is independent of the rotation angle of the part. In contrast, the applied force and effective force are dependent on the rotation angle of the connecting rod. As stated in Section 2, we used a force of 1300 N—slightly larger than the highest measured value in previous studies—in the static analysis of the pedalling scenario. As can be seen in Figure 4a, the load was uniformly distributed over the central part of the pedal’s main body. The highest stress of 33 MPa was located at the contact point with the radial ball bearing (Figure 4b), which is 44% lower than the ultimate tensile strength of the bio-based composite, resulting in a conservative safety factor of approximately 1.8. Consequently, the applied force would be unable to cause material failure. Regarding the deformation, the analysis determined a maximum displacement of 0.29 mm located on the opposite end of the connection between the pedal and the crank (Figure 4c). In contrast, the strain, although small, is focused on the most stressed region which is located at the end where the pedal and connecting rod are joined (Figure 4d). Figure 4e illustrates pedal-to-axle load transmission; as can be seen, the axle played a central role in the mechanical response of the pedal’s main body.

**Figure 5:**
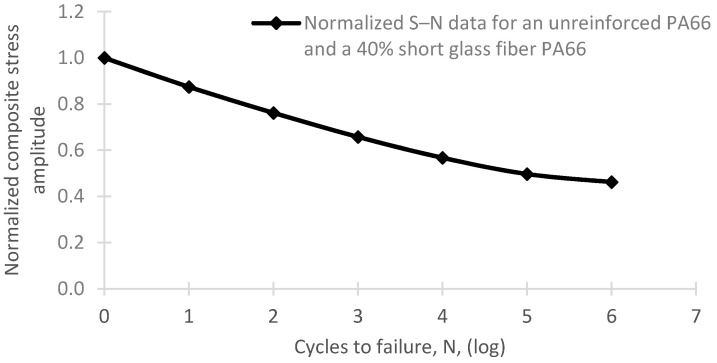
Normalized S-N curve assigned to the PA11 + 60% SGW composite. Adapted from [53].

**Figure 6:**
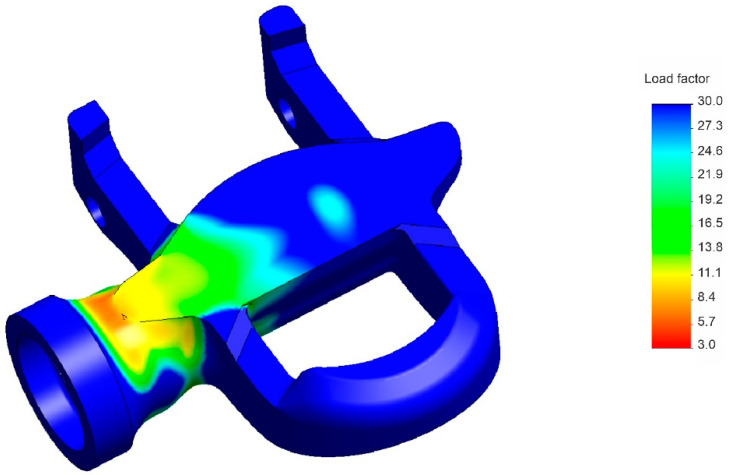
Part load factor due to the simulated cyclical event.

As the load is applied cyclically every pedal stroke, the fatigue behaviour of the pedal’s main body during pedalling is another valuable subject for analysis. This fatigue analysis is actually critical since, at 80 rpm (a typical but conservative pedalling rate), a cyclist can generate 4800 full turns of the connecting rod in an hour. In other words, with the static analysis carried out previously, it would be insufficient to characterize and validate the response of the bio-based composite applied to the geometry of the pedal’s main body. To standardise the fatigue analysis, 10^6^ cycles were applied, with cyclical stresses of constant amplitude, a stress ratio equal to 0, and a single event (SolidWorks parameters). The new finite-element analysis was conducted by applying a load of 165 N to the central portion of the designed pedal’s main body, which is the average pedalling force measured on a mountain stage of the Tour of Italy at a cadence of 80 rpm [52]. As stated in Section 2, the S–N curve used as input for computations on the bio-based material was assumed to be identical with that for PA6 + 40% GF (Figure 5). The S-N curve suggests that after 10^6^ cycles, the mechanical properties of the material will not deteriorate substantially, so it is possible to interpret the results as the material having infinite life. As can be seen in Figure 6, the part withstood 10^6^ cycles with negligible impact, since no point on the mesh has a load factor lower than unity. In fact, the minimum load factor obtained in the analysis is above 5, indicating that the material properties were effectively unchanged. Therefore, it can be concluded that the part would be able to withstand a much higher number of cycles; this is further evidenced by the fact that conservative values were used in the analysis.

### 3.3. Life Cycle Analysis

It is understood that a fully bio-based composite should have a lesser environmental impact than that of a conventional synthetic composite. In this study, the thermoplastic matrix of the bio-based composite was obtained from the castor bean, a ricin oil-rich plant; in addition, the typical high-density (2.48 g/cm^3^) synthetic glass fibre was replaced with a lower density (1.34 g/cm^3^) natural alternative (SGW fibres). As a result, the bio-based material was expected to be more environmentally friendly than the synthetic material. The order of magnitude of the potential gains in environmental impacts was estimated using the sustainability module in SolidWorks 2020, to compare the two types of material through LCA. Like similar commercial software tools, SolidWorks performs weighted computations and does not account for important design and manufacturing characteristics, which limits its usefulness [58]; for this reason, the results of the analysis were considered to be estimates.

The analysis focused on the pedal’s main body and excluded all other elements of the proposed automatic pedal. Table 3 lists the materials used in the simulations. All materials were assumed to be “pure” (i.e., they contained no recycled products). Additionally, the complete life cycle, from the raw materials to the product’s end-of-life, was assumed to occur in Europe. In addition, the pedal was assumed to have a life span of 10 years, to have been manufactured by electrical injection moulding (IM), and to have been carried in a lorry over a distance of 2000 km on average.

Although IM with fully electrical equipment is among the most energy-efficient manufacturing processes [59], its specific energy consumption (SEC) influences the carbon footprint and amount of energy consumed in the overall process and is thus an interesting feature. SEC is influenced by various factors including the nature of the injected material, screw rate, back-pressure, and residence time [60]. In practice, SEC is often taken as 1.47 kWh/kg, which is the average value in the EcoInvest database. Due to the fact that the value for polyamide is known to be greater, we used its value of 1.68 kWh/kg for PA6 in SolidWorks. The choice of SEC for electrical IM is important because SEC is known to be greater for synthetic fibres and pure polymers than it is for composites of lignocellulosic fibres—which have lower melting points and require lower energy to avoid damage during moulding [61]. Therefore, using the previous SEC value would have underestimated the potential of the bio-based composite.

The LCA was performed on the product under the assumption that PA6-based materials have a potential recyclability of 25% and a dumping rate of 51%. This is a favourable assumption for the current glass fibre recyclability, even if recent advances in recycling procedures are considered [10,62]. Based on previous reports [41], the PA11-based materials were assigned 60% recyclability, 30% ashing, and 10% dumping.

As the pedal materials were assumed to be obtained separately, the LCA excluded the energy required for matrix–fibre mixing of the composites. As a result, their overall carbon footprint and energy consumption were underestimated. However, this did not preclude comparing the bio-based and synthetic composites since both were similarly affected in this respect.

**Figure 7:**
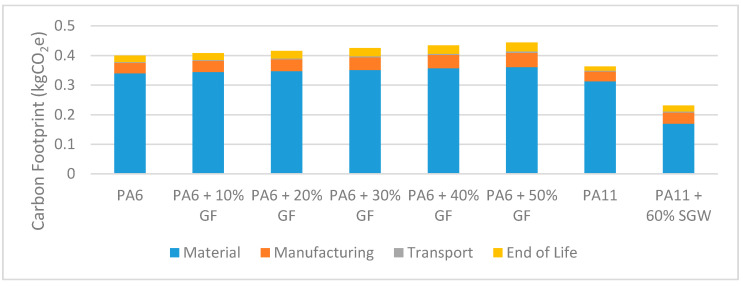
Carbon footprint for a pedal with a 10-year lifespan.

**Figure 8:**
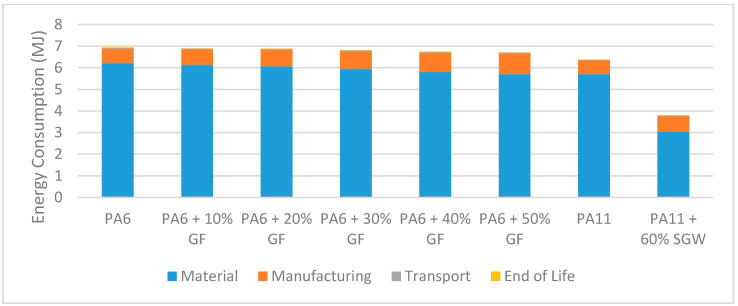
Energy consumption over a 10-year life cycle.

Based on the carbon footprint data in Figure 7 and Table 4, increasing the proportion of reinforcing material in the synthetic composite led to increased CO_2_ emissions; the increase, while moderate, occurred throughout the life cycle (obtainment of the raw material, manufacturing, transport, and end of life). PA6 production has a strong impact on CO_2_ emissions and glass fibre increases the impact slightly. Likely due to the release of various gaseous pollutants during the glass fibre production process, the emission factor of this material is penalised, since the energy consumption is not higher, as seen in Figure 8. In practice, the higher the proportion of glass fibre, the higher the melting point of the composite and the greater the energy needed for plasticization. Glass fibre also increases the viscosity of the resulting composite and requires using a higher injection pressure [61]. In summary, the results of the LCA analysis align with the expected outcomes for the production process: increasing the proportion of glass fibre increased composite density and pedal weight (Table 3), resulting in an increased impact on carbon footprint at the transport and end-of-life stages.

Due to the fact that PA11 is not included in the database of the sustainability module of SolidWorks, and the fact that this software does not allow the creation of new entries, the material was assumed to have the same properties as PA6, and the pedal volume was altered to have the same density as PA11. This was another conservative assumption since PA11 production does not increase atmospheric CO_2_ emissions because the equivalent amount of CO_2_ is absorbed during new plant growth; in addition, the consumption energy for PA11 production is lower than that for any other polyamide [63]. As a result, the LCA significantly overestimated the carbon footprint for the proposed pedal with a bio-based polymer matrix. Even so, as shown in Figure 7, producing a pedal body of bio-based composite is clearly more environmentally sustainable than producing synthetic alternatives.

The energy consumption results of the LCA showed a similar trend to the carbon footprint results. Thus, the bio-based composite was the most environmentally friendly option (see Figure 8 and Table 5), and also the most economical. In fact, production of non-reinforced PA6 monomer accounts for 90% of the entire energy consumption. It should be noted that most of the energy required for IM manufacturing is used to obtain the raw material and manufacture the product, with the former consuming three times more energy than the latter [64]. In our case, producing non-reinforced PA6 would have required almost 10 times more energy than manufacturing the pedal, which indicates the strong influence of the particular polyamide on the energy consumption estimates. Although the results suggest that energy consumption with the synthetic materials decreases as the proportion of fibre increases, the energy of mixing the two components was not considered, so the actual amount of energy to be used would likely increase with an increasing proportion of fibre. Despite this, comparing the energy consumption and carbon footprint results reveals that glass fibre production has a stronger impact on the latter than on the former. This is a result of the increased emission of pollutants in the production process [65] despite the more efficient energy use [66]. In the other LCA stages, for the same reasons as the carbon footprint, energy consumption increased with increasing proportion of glass fibre. Additionally, the low energy consumption of the bio-based composite was due to the decreased proportion of polyamide and the use of a fibre type with lower energy requirements than fibre glass and polyamide. In summary, using the bio-based composite could save approximately 3 MJ with respect to synthetic composites.

The benefit in terms of carbon footprint and energy consumption is significant, in contrast to other recent research in which SGW-reinforced PA11 did not represent a great advantage [41]. It can be reasoned that the environmental benefit of bio-based composites is achieved when they replace technical composites that use matrices such as PA6 or similar. In this sense, if the mechanical properties of the fully bio-based composite were superior, it would be expected that it would replace synthetic materials in more applications. Consequently, it would be advantageous to improve these properties, for example, with the use of reactive coupling agents (from biological origin, if possible).

## 4. Conclusions

The superior material properties of glass fibre-reinforced composites are well known. Depending on the nature of the glass fibres, their size, and their orientation in the thermoplastic matrix, a composite can have good mechanical, chemical, and electrical insulation properties in addition to low weight and cost. However, glass fibres are known to pose serious environmental problems arising from the low recyclability of their widely used composites. In response, scientists are developing new procedures to increase their recycling or replace the reinforcing material for easier reuse. Both areas of research are advancing at a promising pace.

Natural fibres constitute an effective alternative to synthetic raw materials such as glass fibres. Natural fibres are less expensive, lighter, and less dense than synthetic fibres; in addition, they are biodegradable, and hence offer environmental and economic benefits. Mechanically, however, natural fibres are less resistant to impact; they are also more hydrophilic than synthetic materials. These material properties are important for glass fibre-reinforced composites.

In this work, we used finite-element simulations to estimate the mechanical suitability of a bicycle pedal part made from a fully bio-based composite consisting of a natural polyamide (PA11) matrix reinforced with 60 wt% lignocellulosic fibre from SGW. The simulation results indicate the product is mechanically feasible. Based on the nature of the finite-element simulations, it is advisable to assess the performance of the pedal under real-life conditions.

From an environmental standpoint, the preliminary analysis of the life cycle of the product showed considerable benefits in terms of carbon footprint and energy consumed. Specifically, the estimates indicated reductions in the impact of the carbon footprint and energy consumption of more than 40% with the use of the bio-based composite.

Therefore, it is theoretically demonstrated that fully bio-based technical composites can replace synthetic composites in certain applications, and that the environmental benefits can be high. Future improvements in the mechanical properties of PA11 + 60%SGW would enable more widespread use and increase its environmental benefits.

## Figures and Tables

**Table 1:** Tensile and flexural properties of glass fibre-reinforced Polyamide 6 composites and stone-ground wood (SGW)-reinforced Polyamide 11 composites.

**Material**	Tensile Properties			Flexural Properties		
	$\sigma_{t}^{C}$ **(MPa)**	$E_{t}^{C}$ **(GPa)**	$\varepsilon_{t}^{C}\;(\%)$	$\sigma_{f}^{C}\;(\mathrm{MPa})$	$E_{f}^{C}\;(\mathrm{GPa})$	$\varepsilon_{f}^{C}\;(\%)$
PA6	71.2	2.58	68.2	80.3	2.28	6.84
PA6 + 10% GF	92.6	4.76	5.02	140	4.2	-
PA6 + 20% GF	101	5.13	5.41	155	4.92	6.05
PA6 + 30% GF	138	7.92	4.32	207	7.41	4.86
PA6 + 40% GF	166	10.1	3.74	245	9.4	4.65
PA6 + 50% GF	195	13.9	3.06	303	12.9	3.79
PA11	38.3	1.4	25.0	40.0	0.9	7.4
PA11 + 60% SGW	59.6	5.8	2.8	102.7	4.1	3.2

**Table 2:** Ranges spanned by the tensile and flexural strength properties of the materials.

Material	Tensile Properties		Flexural Properties	
	*n* ^1^	$\left[\sigma_{0}^{C}-\sigma_{n}^{C}]\;(\mathrm{MPa}\right)$	*n* ^1^	$\left[\sigma_{0}^{C}-\sigma_{n}^{C}]\;(\mathrm{MPa}\right)$
PA6 + 10% GF	54	51.7–170	167	13.8–300
PA6 + 20% GF	90	60–195	348	50–300
PA6 + 30% GF	211	50–193	676	40–800
PA6 + 40% GF	259	65–470	214	115–352
PA6 + 50% GF	202	90–605	168	175–840

^1^*n* is the number of plastic grades per composites and intervals considered by the materials library examined [43].

**Table 3:** Selected properties of pedals consisting of various composites.

Property	Density (g/cm^3^)	Weight (g)	Resin Volume (%)	Fibre Volume (%)
PA6	1.12	33.17	100	-
PA6 + 10% GF	1.18	35.09	95.22	4.78
PA6 + 20% GF	1.26	37.25	89.86	10.14
PA6 + 30% GF	1.34	39.70	83.78	16.22
PA6 + 40% GF	1.43	42.49	76.86	23.14
PA6 + 50% GF	1.54	45.70	68.89	31.11
PA11	1.03	30.50	100	-
PA11 + 60% SGW	1.20	35.42	46.45	53.50

**Table 4:** Carbon footprint (kg CO_2_) by composite and life cycle analysis (LCA) stage with a life expectancy of 10 years.

Stage	PA6	PA6 + 10% GF	PA6 + 20% GF	PA6 + 30% GF	PA6 + 40% GF	PA6 + 50% GF	PA11	PA11 + 60% SGW
Material	0.340	0.344	0.347	0.351	0.357	0.361	0.313	0.170
Manufacture	0.035	0.038	0.040	0.043	0.045	0.048	0.033	0.038
Transport	0.003	0.003	0.004	0.004	0.004	0.004	0.003	0.004
End of life	0.022	0.023	0.025	0.027	0.028	0.030	0.014	0.020
Total	0.400	0.408	0.415	0.425	0.434	0.443	0.363	0.231

**Table 5:** Energy consumption (MJ) by LCA stage for various materials with a life expectancy of 10 years.

Stage	PA6	PA6 + 10% GF	PA6 + 20% GF	PA6 + 30% GF	PA6 + 40% GF	PA6 + 50% GF	PA11	PA11 + 60% SGW
Material	6.200	6.117	6.058	5.933	5.800	5.700	5.700	3.036
Manufacture	0.673	0.712	0.756	0.805	0.862	0.927	0.619	0.702
Transport	0.046	0.049	0.052	0.056	0.060	0.064	0.043	0.052
End of life	0.017	0.018	0.019	0.020	0.022	0.022	0.011	0.012
Total	6.936	6.896	6.885	6.814	6.744	6.713	6.373	3.802

## Data Availability

There is no additional data.
